# Cinobufotalin regulates the USP36/c-Myc axis to suppress malignant phenotypes of colon cancer cells *in vitro* and *in vivo*

**DOI:** 10.18632/aging.205661

**Published:** 2024-03-15

**Authors:** Yongjun Hu, Ming Luo

**Affiliations:** 1 Department of General Surgery, The Second Xiangya Hospital, Central South University, Changsha 410011, China

**Keywords:** colon cancer, USP36, oxaliplatin, c-Myc, cinobufotalin

## Abstract

Ubiquitin-specific protease 36 (USP36) has been reported to exhibit oncogenic effects in various malignancies, but the function of USP36 in colon cancer progression remains indefinite. Herein, we aimed to determine the role and mechanism of USP36 in malignant phenotypes of colon cancer cells and explore the potential drug targeting USP36. Bioinformatics analyses indicated that USP36 is highly expressed and significantly related to tumor stages in colon cancer. Besides, USP36 was further up-regulated in oxaliplatin (Oxa)-resistant colon cancer cells. Colony formation, Edu staining, Transwell, wound healing, sphere formation, and CCK-8 assays were conducted and showed that the proliferation, Oxa-resistance, migration, stemness, and invasion of HCT116 cells were promoted after overexpressing USP36, while suppressed by USP36 knockdown. Mechanically, USP36 enhances c-Myc protein stabilization in HCT116 cells via deubiquitination. AutoDock tool and ubiquitin-AMC hydrolysis assay identified cinobufotalin (CBF), an anti-tumor drug, maybe a USP36 inhibitor by inhibiting its deubiquitination activity. CBF significantly prohibited proliferation, migration, invasion, and stemness of HCT116 cells and reversed Oxa-resistance, whereas enforced expression of USP36 blocked these effects. Moreover, *in vivo* analyses confirmed the oncogenic role of USP36 and the therapeutic potential of CBF in the malignancy of colon cancer. In conclusion, CBF may be a promising therapeutic agent for colon cancer due to its regulation of the USP36/c-Myc axis.

## INTRODUCTION

Colon cancer is the fifth most common malignancy with an estimated 1.15 million new cases worldwide in 2020 [1]. Despite the advances in detection and treatment over the past decade, colon cancer is the third leading cause of cancer death in the world, representing 5.5% of mortality, which remains a vital public health issue [1]. Among all diagnosed colon cancer, more than half of them have spread to surrounding tissues or distant parts of the body, which exhibit a higher rate of metastasis and recurrence [2], thereby leading to a poor prognosis [3]. Endoscopic or surgical resection is not an optimal choice for late-stage colon cancer [4]. Meanwhile, multidrug resistance has become the most challenging obstacle impeding the success of both radiotherapy and chemotherapy [5]. Therefore, uncovering novel molecular mechanisms related to carcinogenesis as well as the progression of colon cancer can aid the development of novel effective therapeutic targets for this malignancy, which is critical to improving the survival rates of patients with colon cancer.

In the last two decades, the critical role of post-translational modifications (PTMs), such as phosphorylation, methylation, as well as acetylation, in the pathogenesis of cancer has grabbed the attention from researchers around the world [6]. As two mutually reversible PTMs, ubiquitylation and de-ubiquitylation play many critical regulatory roles in multiple cell physiological and pathological processes, which include protein stabilization, metabolism, and localization [7, 8]. Ubiquitylation is a process that proteins are tagged with ubiquitin and destined for degradation, which could be reversed by releasing ubiquitin from proteins with deubiquitinating enzyme (DUB) [9]. The ubiquitin-specific proteases (USP) family, the largest DUB family containing 60 proteases, has been demonstrated to be involved in either impairing the tumor suppressor gene activity or strengthening the functions of oncogenes in many cancers, such as colon cancer [10, 11]. For example, USP28 has been demonstrated to help c-Myc stabilization, thereby contributing to the initiation of colorectal cancer [12]. Research has proved that USP5 promoted the growth and chemoresistance of colorectal cancer cells, which relied on its de-ubiquitylation on several oncogenes [13]. Evidence suggests that the USP39 protein contributes to not only the growth but also the metastasis of colorectal cancer [14]. As the emerging oncogenic role of USPs in multiple cancers, agents targeting USP has become a promising therapeutic strategy for patients with cancer.

Based on the bioinformatics online database, USP36 was shown a more significant up-regulation in colon cancer than that in normal samples. However, the role and mechanisms behind the abnormal expression of USP36 in colon cancer remain elusive. Hence, our present study attempts to determine the role of USP36 in malignant phenotypes of colon cancer cells and uncover the function mechanism and further explored potential therapeutic drugs targeting USP36 for patients with colon cancer. The experimental design of this study was described in Supplementary Figure 1.

## RESULTS

### USP36 is highly expressed in colon cancer and further up-regulated in Oxa-resistant colon cancer cells

**Figure 1:**
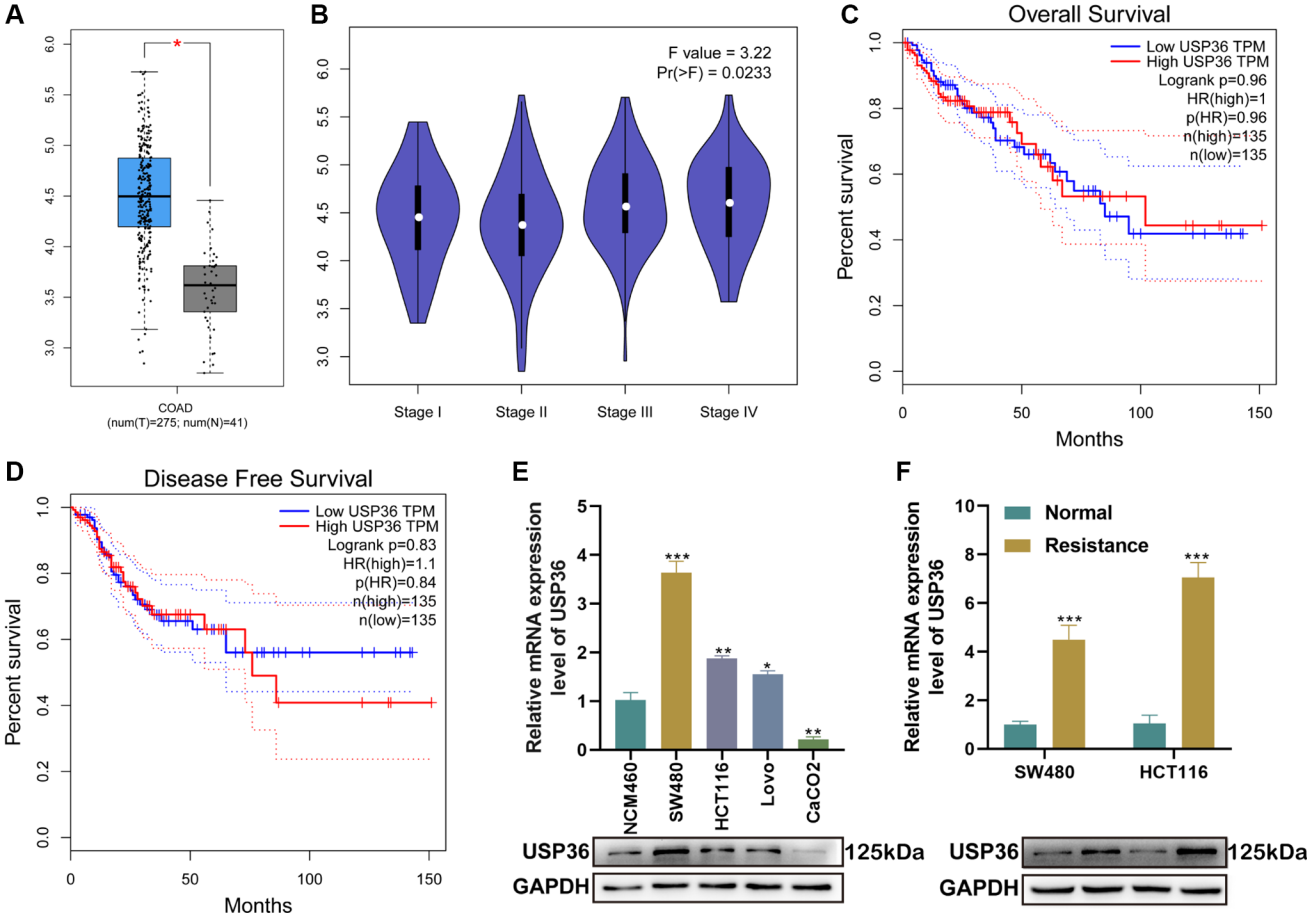
**USP36 is highly expressed in colon cancer and further up-regulated in Oxa-resistant colon cancer cells.** (**A**) The expression profile of USP36 between COAD and normal samples. (**B**) The expression profile of USP36 in different stages of COAD patients. (**C**) The analysis between OS and USP36 expression in COAD patients. (**D**) The analysis between DFS and USP36 expression in COAD patients. (**E**) The mRNA (top) and protein (button) expression levels of USP36 in NCM460 cells and colon cancer cells (SW480, HCT116, Lovo, and CaCO2). (**F**) The mRNA (top) and protein (button) expression levels of USP36 between Oxa-resistant and the parental colon cancer cells. ^*^p < 0.05, ^**^p < 0.01, and ^***^p < 0.005.

Based on the analysis from GEPIA, the expression of USP36 in colon cancer samples was significantly higher than that in the normal (Figure 1A), and was closely related to the tumor stage (Figure 1B). However, both OS and DFS analysis indicated there is no significant correlation between the USP36 expression and the survival times of patients with colon cancer (Figure 1C, 1D). In addition, the expression level of USP36 was monitored between NCM460 (the normal human colon mucosal epithelial cells) and colon cancer cells (SW480, HCT116, Lovo, and CaCO2), and between Oxa-resistant cell lines and the parental one. Compared with NCM460 cells, USP36 was highly expressed in all colon cancer cell lines and further up-regulated in Oxa-resistant colon cancer cells (Figure 1E, 1F).

### USP36 overexpression aggravates proliferation, Oxa-resistance, stemness, migration, and invasion of colon cancer cells *in vitro*

**Figure 2:**
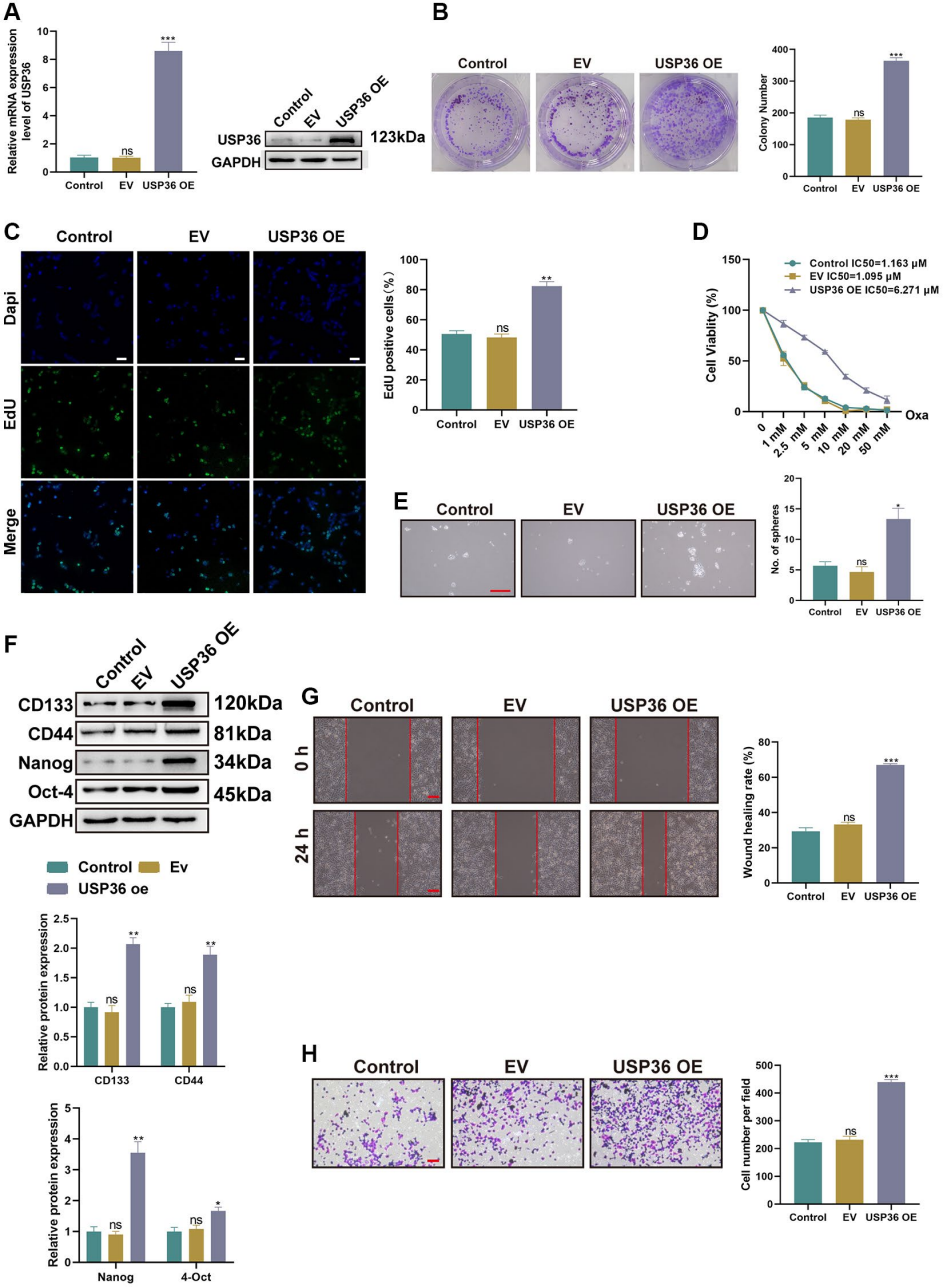
**USP36 overexpression aggravates malignant phenotypes of colon cancer cells *in vitro*.** (**A**) The mRNA (top) and protein (button) expression levels of USP36 in HCT116 cells were respectively detected by RT-PCR and western blot to validate the transfection efficiency of USP36 OE. (**B**, **C**) The cell proliferation was detected by colony formation (**B**) and Edu staining (C, Scalebar=100 μm) assays. (**D**) The cell viability was detected by CCK-8 assay after a series of concentrations of Oxa for the determination of IC50 value to Oxa. (**E**) The cellular self-renewal capacity was investigated by sphere formation assay (Scalebar = 100 μm). (**F**) The protein expression of cancer stem cell-related markers (CD133, CD44, Nanog, and Oct-4) was detected by western blot. (**G**) The cell migration was detected by wound healing assay (Scalebar = 100 μm). (**H**) The cell invasion was observed by Transwell assay (Scalebar = 100 μm). ^*^p < 0.05, ^**^p < 0.01, and ^***^p < 0.005; Abbreviation: ns: none significance.

To uncover the role of USP36 in the malignant phenotypes of colon cancer cells, HCT116 cells were transfected with either the USP36 OE plasmids or empty controls (EV). The transfection of USP36 OE led to a significant increase of USP36 expression in HCT116 cells at both mRNA and protein levels (Figure 2A, 2B), confirming the successful overexpression by transfection. The proliferative ability of HCT116 cells was significantly strengthened after the transfection of USP36 OE, as indicated by the colony formation and Edu staining assays (Figure 2C, 2D). CCK-8 assay indicated that the IC50 values to Oxa of HCT116 cells in control, EV, and USP36 OE groups were 1.095 μM, 1.163 μM, and 6.271 μM, respectively (Figure 2E), suggesting that the up-regulation of USP36 contributes to the Oxa-resistance of HCT116 cells. To determine whether USP36 is also related to the stem cell characteristic of self-renewal, the population of stem cells residing in HCT116 cells with or without different transfections was assessed by sphere formation analysis. The results showed that USP36 overexpressing HCT116 cells exerted stronger sphere-forming ability (Figure 2F), which demonstrated the contributive role of USP36 in cancer stemness. Similarly, western blot assay revealed that the expression levels of stem cell markers (CD133, Nanog, Oct-4, and CD44) in USP36 overexpressing HCT116 cells were higher than those in the control HCT116 cells (Figure 2F). In addition, both the migration and invasive capabilities of HCT116 cells were significantly strengthened by the transfection of USP36 OE, as revealed by wound healing and Transwell assays (Figure 2G, 2H). Collectively, a series of functional experiments revealed the oncogenic effect of USP36 on colon cancer cells *in vitro*.

### USP36 contributes to the tumor stemness, growth, Oxa-resistance, and metastasis of colon cancer in xenograft models

**Figure 3:**
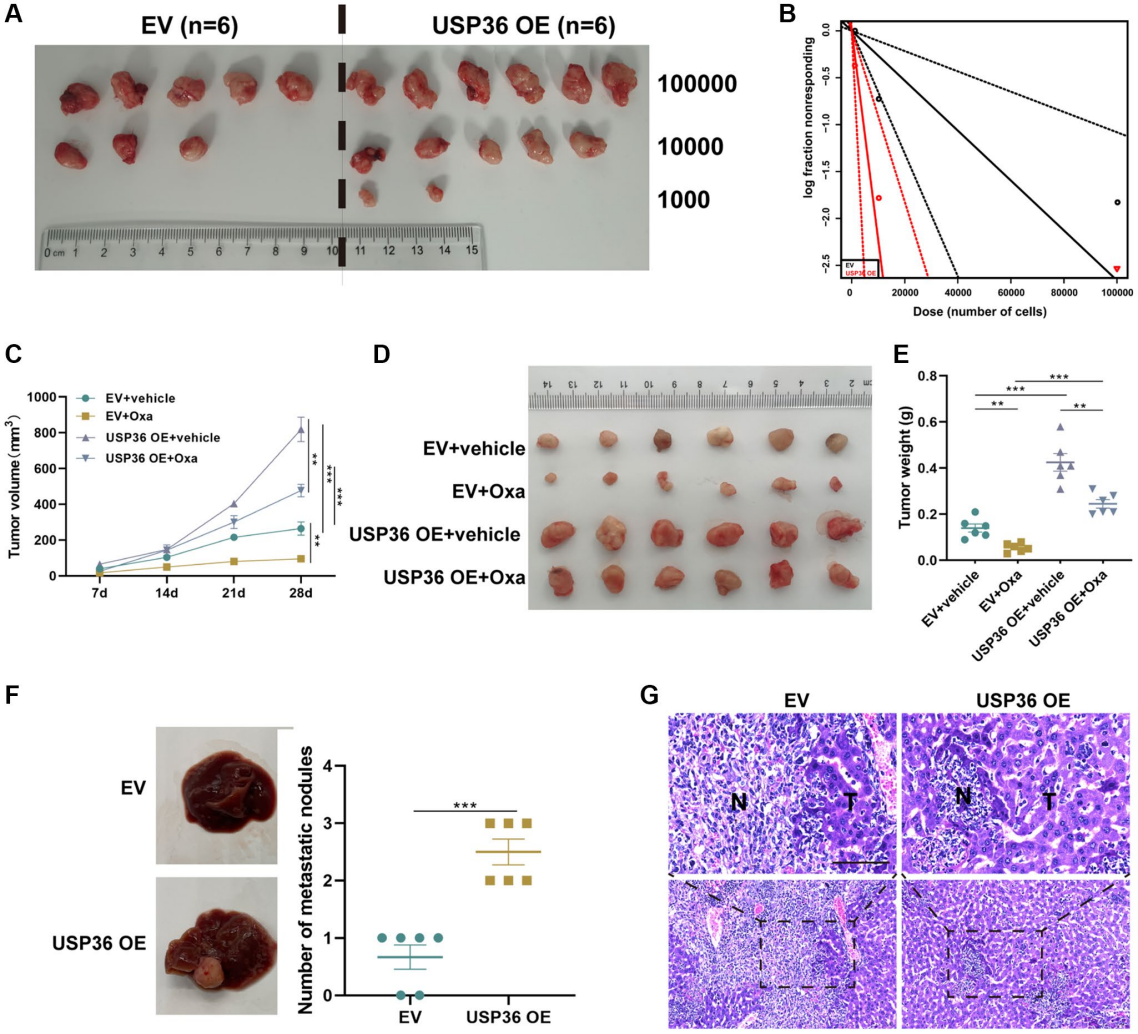
**USP36 overexpression promotes the stemness, growth, Oxa-resistance, and metastasis of colon cancer.** (**A**) The xenograft formation of 1,000, 10,000, and 100,000 HCT116 cells transfected with EV or USP36 OE. (**B**) Tumorsphere formation frequency of HCT116 cells was calculated by ELDA. (**C**) Tumor growth curve. (**D**) The tumor was harvested from each group on day 28. (**E**) Weight of the harvested tumor. (**F**) Representative of gross view (left) and the number of metastatic nodules (right) of the liver from the metastatic murine model. (**G**) H&E staining for the liver tissues from the metastatic murine model (Scale bar = 100 μm). ^**^p < 0.01 and ^***^p < 0.005.

Next, the oncogenic role of USP36 in colon cancer was validated by *in vivo* animal studies. The limiting dilution experiment was carried out to investigate the relation between USP36 and TIC frequency. After the inoculation of three different dilutions (100,000, 10,000, and 1,000 cells) of HCT116 cells with EV or USP36 OE transfection for 4 weeks, tumors were harvested for ELDA analysis (Figure 3A, 3B). The result showed that USP36 overexpressing HCT116 cells exhibited significantly higher stem cell frequency in comparison of HCT116 cells (Table 1). Tumors formatted by USP36 overexpressing HCT116 cells showed a faster growth rate and less sensitivity to Oxa, when compare with HCT116 control cells (Figure 3C–3E). The analysis on liver metastasis murine revealed that the overexpression of USP36 caused a promotion of the liver metastasis of HCT116 cells (Figure 3F). H&E staining results showed that liver sections in the USP36 OE group exhibit the highest number and largest area of cancerous nests in the liver (Figure 3G). These results collectively revealed that USP36 was responsible for the cancer stemness, tumor growth, Oxa-resistance, as well as liver metastasis in colon cancer, which may be a promising therapeutic target for treating patients with colon cancer.

**Table 1:** The confidence intervals for 1/(stem cell frequency).

**Cell number**	**Empty vector**	**USP36 overexpression**
1000	6/0	6/2
10000	6/3	6/5
100000	6/5	6/6
1/(stem cell frequency) (95% Confidence intervals)	37586	4479
*P*-value	0.000675	

### c-Myc is a direct target of USP36 in colon cancer cells

**Figure 4:**
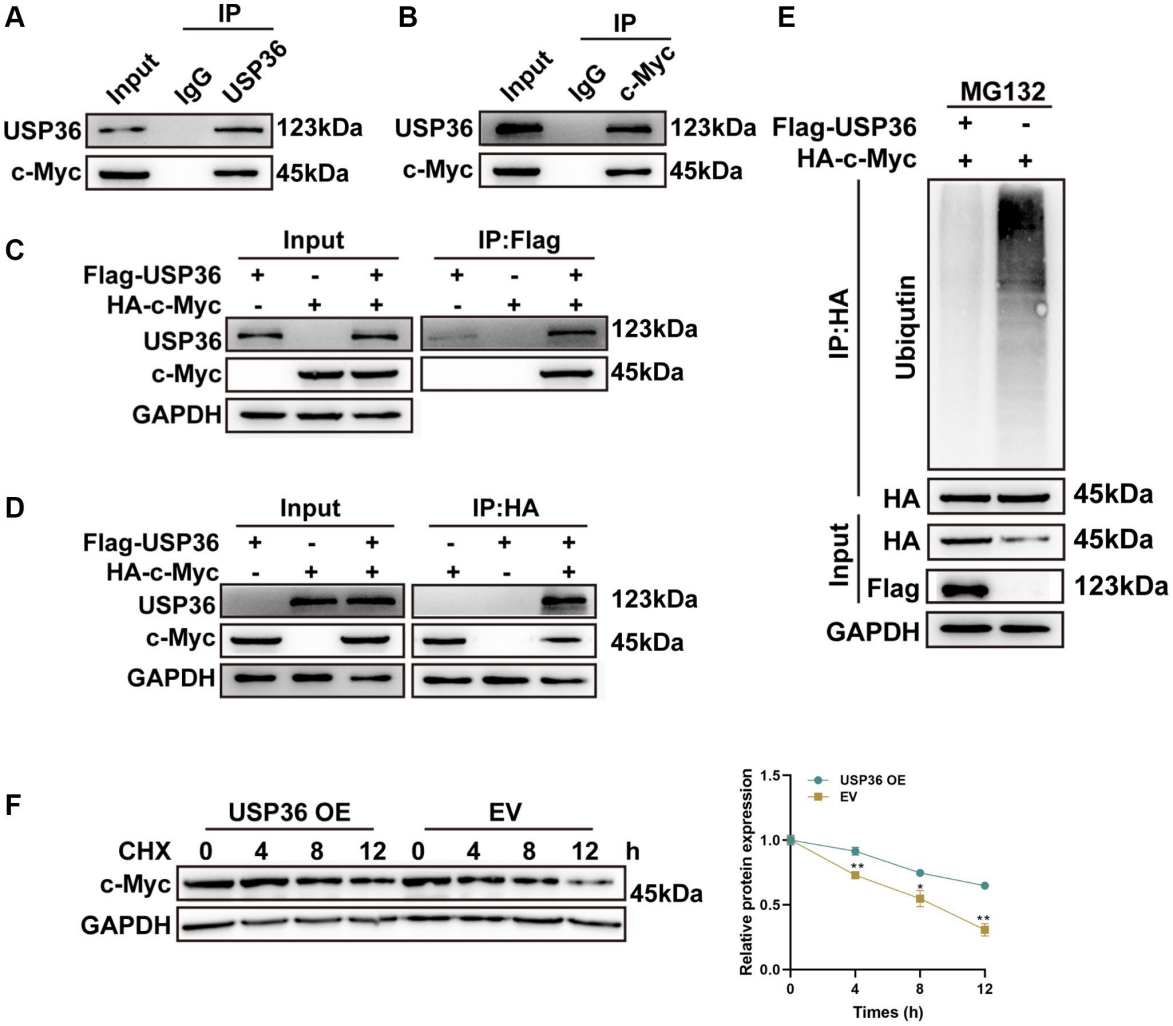
**USP36 promotes c-Myc expression via its deubiquitinating role.** (**A**) Co-IP assays of USP36 in HCT116 cells. (**B**) Co-IP assays of c-Myc in HCT116 cells. (**C**) Co-IP assays of Flag in HCT116 cells transfected with Flag-USP36 or HA-c-Myc or their combination. (**D**) Co-IP assays of HA in HCT116 cells transfected with Flag-USP36 or HA-c-Myc or their combination. (**E**) *In vitro* ubiquitination assay for HCT116 cells with the transfection of HA-c-Myc combined with or without Flag-USP36. (**F**) CHX half-life assay. ^*^p < 0.05 and ^**^p < 0.01.

The proto-oncogene c-Myc plays a critical role in multiple malignancies, including colon cancer [15]. USP36 has been identified as a novel DUB of c-Myc to promote its stabilization [16]. Hence, the interaction between USP36 and c-Myc in colon cancer cells was investigated to uncover the potential mechanism underlying the oncogenic effect of USP36. The Co-IP assays with anti-USP36 and anti-c-Myc on lysates of HCT116 cells collectively confirmed the endogenous interaction of USP36 and c-Myc proteins (Figure 4A, 4B). In the meantime, the exogenous Co-IP assays (Figure 4C, 4D) showed similar results, corroborating that USP36 directly interacts with the c-Myc protein. Whether USP36 regulates c-Myc protein stability was subsequently explored. As shown in Figure 4E, the ubiquitin conjugation to c-Myc was significantly suppressed in HCT116 cells after overexpressing USP36. Meanwhile, overexpressing USP36 effectively delayed the c-Myc protein degradation of HCT116 cells (Figure 4F). Therefore, it could suppose that USP36 contributes to the stabilization of the c-Myc protein in colon cancer cells via deubiquitination, which may be the mechanism behind the promotive function of USP36 on the aggressive phenotypes of colon cancer cells.

### CBF interacts with USP36 and inhibits its enzyme activity to promote the ubiquitination and degradation of c-Myc

**Figure 5:**
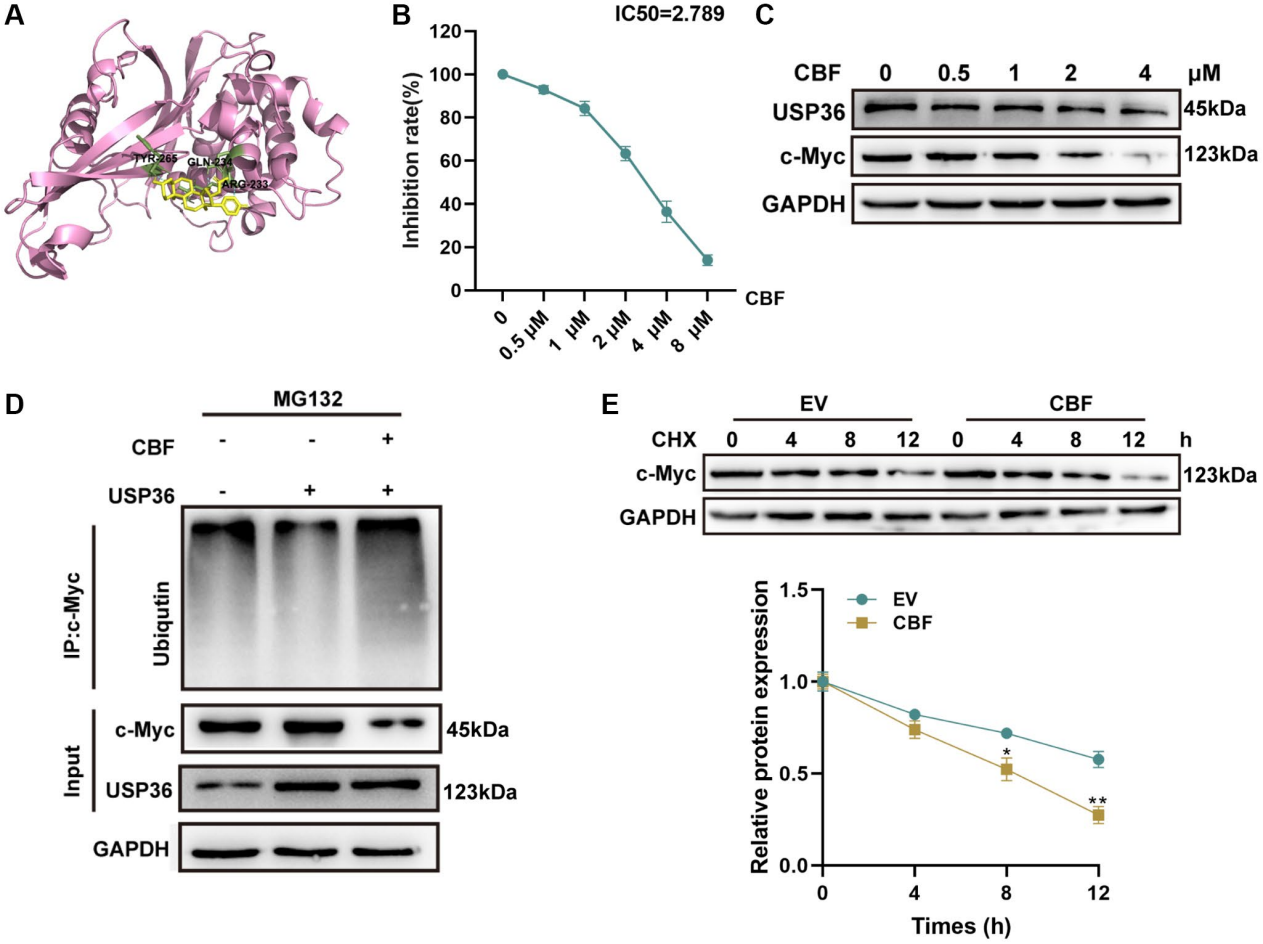
**CBF interacts with USP36 and inhibits its enzyme activity to promote the ubiquitination and degradation of c-Myc.** (**A**) Molecular docking diagram of CBF with USP36. (**B**) The inhibition of CBF on USP36 activity was determined by Ub-AMC hydrolysis assay. (**C**) The protein expression of USP36 and c-Myc in HCT116 cells with different doses of CBF was detected by western blot. (**D**) *In vitro* ubiquitination assay for HCT116 cells with the transfection of USP36 OE combined with or without CBF treatment. (**E**) CHX half-life assay. ^*^p < 0.05 and ^**^p < 0.01; Abbreviation: ns: no significance.

Based on the molecular docking analysis, CBF (Supplementary Figure 2), a potential drug against other cancers [17, 18], was identified to interact with the catalytic domain of USP36 (Figure 5A). Hence, the effect of CBF on USP36 activity and c-Myc expression was subsequently explored. Ub-AMC hydrolysis assay showed that CBF suppressed USP36 activity in a dose-dependent way (IC50 = 2.75 μM) (Figure 5B). Under the intervention of CBF, c-Myc protein expression levels were obviously decreased with increasing CBF concentration (Figure 5C). Moreover, the ubiquitination level of c-Myc was reduced by overexpressing USP36 in HCT116 cells, and this effect was blocked by CBF (Figure 5D). Similarly, the CHX half-life assay showed the intervention of CBF plays a promotive role in the degradation of the c-Myc protein (Figure 5E). Collectively, CBF reduces c-Myc stabilization by impairing the deubiquitination function of USP36.

### Suppressive effect of CBF on colon cancer cells is mediated by USP36

**Figure 6:**
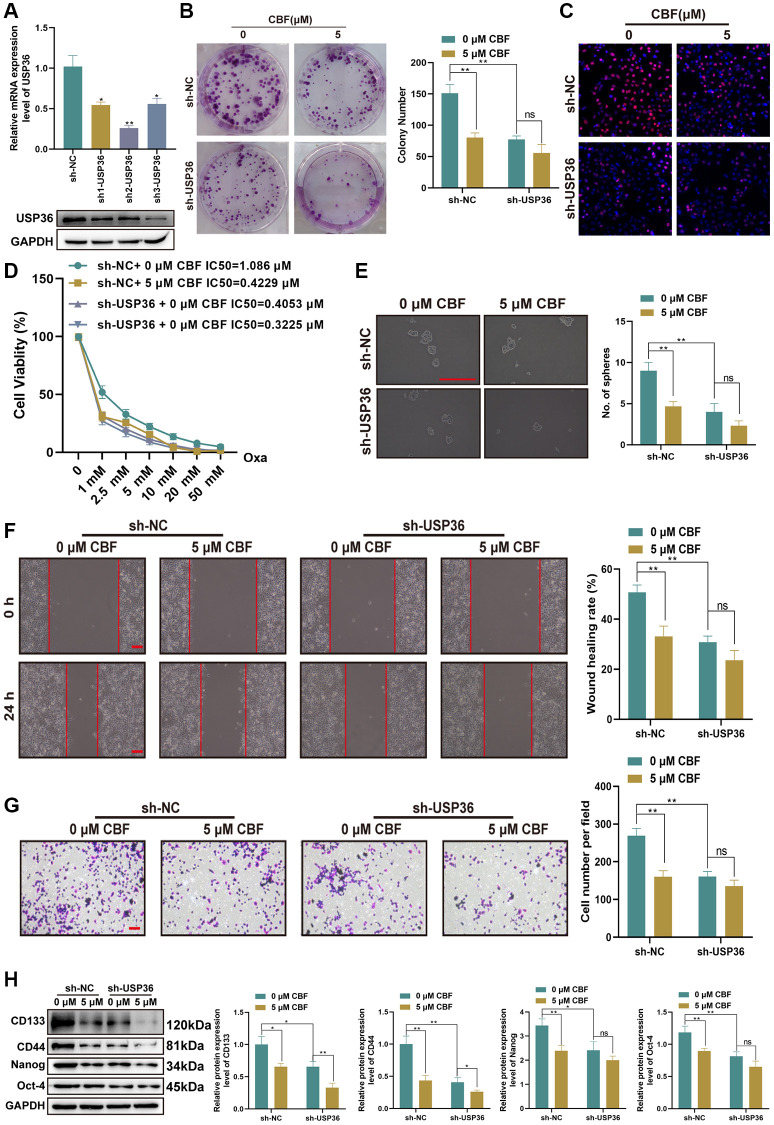
**Suppressive effect of CBF on malignant phenotypes of colon cancer cells is mediated by USP36.** (**A**) The mRNA (top) and protein (button) expression levels of USP36 in HCT116 cells were respectively detected by RT-PCR and western blot to validate the transfection efficiency of sh-USP36. (**B**, **C**) The cell proliferation was detected by colony formation (**B**) and Edu staining (**C**, Scale bar = 100 μm) assays. (**D**) The cell viability was detected by CCK-8 assay after a series of concentrations of Oxa for the determination of IC50 value to Oxa. (**E**) The cellular self-renewal capacity was investigated by sphere formation assay (Scale bar=100 μm). (**F**) The protein expression of cancer stem cell-related markers (CD133, CD44, Nanog, and Oct-4) was detected by western blot. (**G**) The cell migration was detected by wound healing assay (Scale bar = 100 μm). (**H**) The cell invasion was observed by Transwell assay (Scale bar = 100 μm). ^*^p < 0.05 and ^**^p < 0.01; Abbreviation: ns: no significance.

To explore the effect of CBF on colon cancer cells and determine whether its effect is related to USP36, HCT116 cells were treated with or without CBF after the transfection of sh-NC or sh-USP36, and subjected to functional biological detections. The transfections of sh1-USP36, sh2-USP36, and sh3-USP36 effectively down-regulated the expression of USP36 expression levels (Figure 6A, 6B). Among them, sh2-USP36 displayed the highest efficiency (Figure 6A, 6B), so sh2-USP36 was chosen for the subsequent experiments. For HCT116 cells with the transfection of sh-NC, it was found that CBP treatment (5 μM) significantly impairs their proliferation, ability against Oxa, stemness, migration, as well as invasion (Figure 6C–6H). However, in USP36 silencing HCT116 cells, there is no significant difference in cell proliferation, Oxa resistance, stemness, migration, and invasion between the treatment with 0 and 5 μM CBF (Figure 6C–6H). These results supported the anti-colon cancer effect of CBF and strongly suggested that the suppressive effect of CBF on malignant phenotypes of colon cancer cells is mediated by USP36.

### CBF inhibits colon tumor growth and metastasis and enhances the anti-tumor effect of Oxa *in vivo*

**Figure 7:**
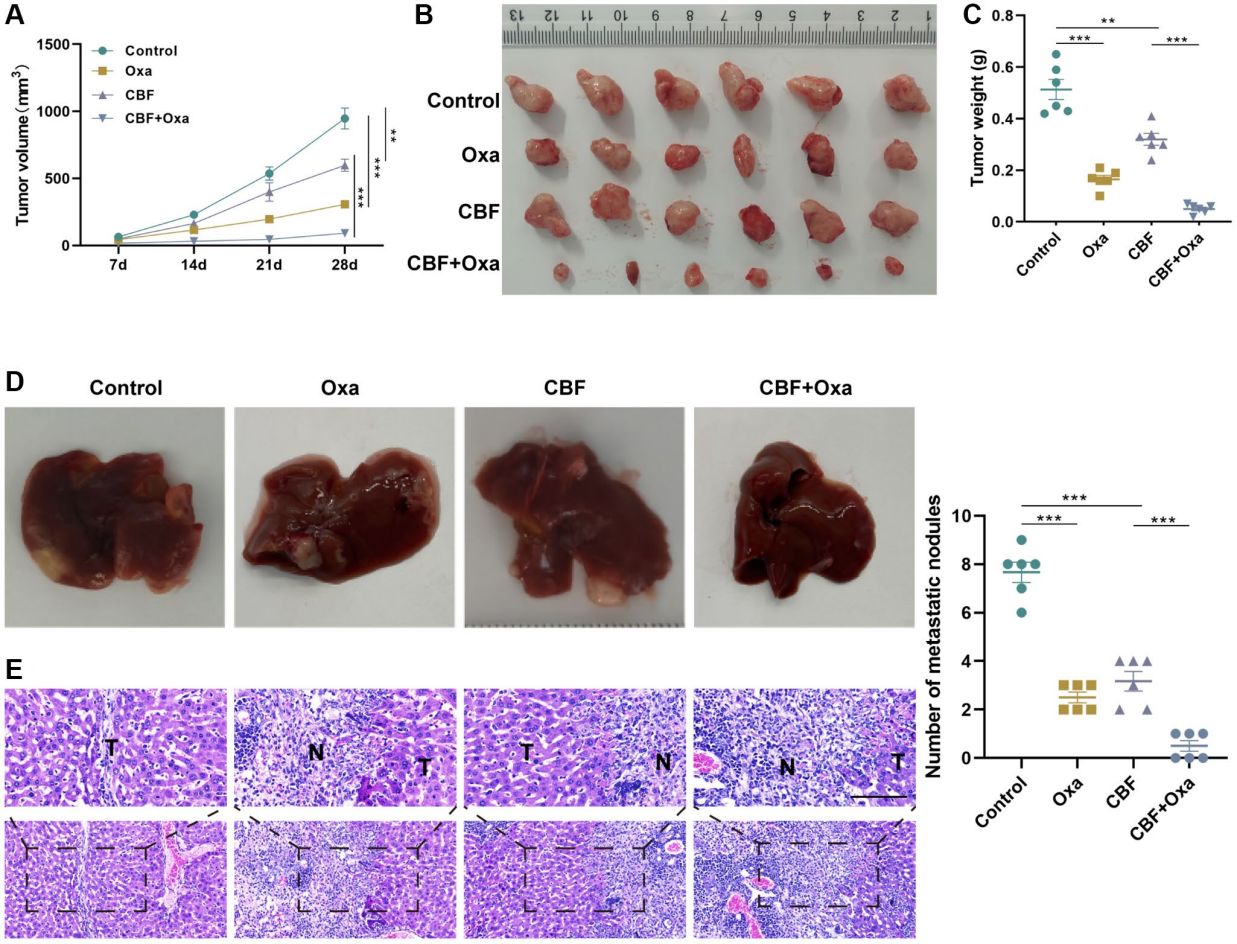
**CBF inhibits colon tumor growth and metastasis, and enhances the anti-tumor effect of Oxa *in vivo*.** (**A**) Tumor growth curve. (**B**) The tumor was harvested from each group on day 28. (**C**) Weight of the harvested tumor. (**D**) Representative of gross view (left) and the number of metastatic nodules (right) of the liver from the metastatic murine model. (**E**) H&E staining for the liver tissues from the metastatic murine model (Scale bar = 100 μm). ^**^p < 0.01 and ^***^p < 0.005.

Lastly, the anti-tumor role of CBF was further confirmed *in vivo*. The tumor growth curve showed that the CBF group developed significantly smaller tumors than the control group (Figure 7A, 7B), verifying that CBF effectively suppresses colon tumor growth, which was corroborated by the tumor weight result (Figure 7C). Additionally, in comparison with the control group, the CBP group exhibited fewer metastatic nodules and aggressive lesions in the liver (Figure 7D, 7E). Consistent with the results of *in vitro* analyses, *in vivo* studies revealed that CBF intervention could significantly enhance Oxa efficacy in colon tumors (Figure 7A–7E). Meanwhile, in the liver metastasis model mice, it was observed that the combination of CBF and Oxa significantly suppressed the tumor metastasis as demonstrated by the decreased number of metastatic nodules and H&E staining results in the liver (Figure 7D, 7E). All these results indicated that CBF could be a promising anti-neoplastic agent for enhancing chemosensitivity upon Oxa treatment.

## DISCUSSION

Although the huge progress has been achieved in the last two decades, colon cancer remains a highly lethal malignancy [19]. Aggressiveness, chemoresistance, and metastasis are the primary causes of mortalities in colon cancer [20]. It was gradually accepted that the high rate of relapse and even resistance of cancer to chemotherapy is attributed from cancer stem cells (CSCs) [21]. In various types of solid tumors, including colon cancer, CSCs have been identified, which not only contributes to tumorigenesis and tumor heterogeneity but also to metastasis and drug resistance despite it constitutes only a small fraction of the total cancer cell population [22]. Therefore, targeting CSCs is an important way to develop novel therapeutic strategies for improving the prognosis of patients with colon cancer [23].

Based on accumulating evidence, DUB plays an undisputed essential role in the maintenance of pluripotency of CSCs during cancer development [24]. For example, USP54 is found to be overexpressed in colorectal cancer, which confers stem-cell-like traits to colorectal cancer cells and facilitates intestinal tumorigenesis [25]. USP22 strengthens the tumorigenic activity of breast cancer cells by promoting c-Myc (a CSC-associated transcription factor) stabilization [26]. Moreover, several DUBs, such as USP28 [27] and USP8 [28], have been reported to regulate hypoxia-inducible factor proteins, the key mediators in maintaining multiple CSC populations under the hypoxic tumor microenvironment [29]. It has been shown that USP36 is crucial in ovarian cancer development due to its direct deubiquitylation for PrimPol, of which overexpression correlates with poor prognosis [30]. More recently, USP36 facilitates the development of glioblastoma through mediating ALKBH5 protein stability [31]. In our present study, we found an up-regulation of USP36 in colon cancer based on the bioinformatic database-GEPIA. Nevertheless, few reports have shown the relationship between USP36 and the aggressive phenotypes of colon cancer.

Given the oncogenic activity of USP36 has been demonstrated in various malignancies, we wonder whether USP36 is related to the aggressiveness, chemoresistance, and metastasis of colon cancer and could be a promising target of colon cancer management for improving prognosis. Our research provides multiple lines of evidence for the oncogenic role of USP36 in colon cancer cells. *In vitro* experiments indicated that the proliferation, migration, stemness, and invasion of colon cancer cells were all promoted after overexpressing USP36, while were impaired by silencing USP36. As a third-generation platinum-based anticancer agent, Oxa is the first-line chemotherapy commonly used for colon cancer; but many patients usually occur resistance to Oxa after long-term use, resulting in treatment failure [32]. Our data also indicated that USP36 contributed to the Oxa resistance to colon cancer cells, as indicated by the IC50 value of HCT116 cells to Oxa was significantly changed after the transfection of USP36 OE or sh-USP36. Moreover, *in vivo* studies further validated the oncogenic role of USP36 in colon cancer.

To explore the potential drug targeting USP36 for colon cancer treatment, the mechanism underlying the regulation of USP36 on the aggressive phenotypes of colon cancer cells was also revealed in this study. As a transcription factor, c-Myc is closely related to the regulation of different cancer cellular functions, such as cell survival, cellular proliferation, and metabolic reprogramming [33, 34]. The aberrant up-regulation of c-Myc is commonly found in 70% of colon cancer, supporting that c-Myc is an important driver of colon cancer progression [15]. For colon cancer stem cells, knocking down c-Myc in cells caused suppression in cell invasion and migration [35]. Moreover, c-Myc up-regulation was observed in surviving tumor cells after platinum-based chemotherapy [36]. It has been revealed that c-Myc is a direct target of USP36, which could be deubiquitylated by USP36 to maintain its stabilization [16, 37]. Similarly, our data showed that USP36 interacted with c-Myc, and overexpression of USP36 significantly blocked ubiquitin-mediated degradation of c-Myc in HCT116 cells. These results demonstrated that USP36 drove the aggressive phenotypes of colon cancer cells by reducing c-Myc ubiquitination and degradation, thereby aggravating cancer progression.

Due to its oncogenic function in colon cancer, the USP36/c-Myc axis may be a potential target for developing the anticancer drug. CBF, a cardiotonic steroid extracted from dried toad venom, exerts antitumor activity and has the ability to enhance the chemotherapeutic effect in several cancers [38, 39]. However, whether CBF plays an anti-cancer function in colon cancer remains unknown. Here in this study, based on the AutoDock tool, CBF was identified as a USP36 inhibitor by targeting the catalytic domain of USP36. The binding of CBF would then directly affect the recognition of ubiquitin substrate by USP36, thereby reducing its enzymatic activity. This was verified by the Ub-AMC hydrolysis assay, which showed that CBF inhibits the enzymatic activity of USP36 with an IC50 at 2.75 μM. Notably, the stabilization of c-Myc promoted by USP36 overexpression in HCT116 cells was blocked after CBF intervention.

Finally, we further explored the potential of CBF against colon cancer. Our data demonstrated that CBF significantly suppressed proliferation, stemness, invasion, as well as migration of colon cancer cells and sensitizes these cells to Oxa-induced cytotoxicity, whereas enforced expression of USP36 partly blocked these effects. More importantly, *in vivo* assays further indicated that CBF could not only effectively inhibit colon tumor growth and metastasis but also significantly improve Oxa efficacy in colon tumors, which supported the promising potential of CBF as an emerging therapeutic for colon cancer.

In summary, our research demonstrated that USP36 promotes the aggressiveness, chemoresistance, and metastasis of colon cancer by deubiquitinating and stabilizing c-Myc, and uncovered a potential USP36 inhibitor, CBF, in the treatment of colon cancer. However, several limitations remain to this study. First, the specific mechanism underlying how CBF influences USP36 activity remains unclear. Besides, exploring the effect of CBF in orthotopic models of colon cancer is necessary to further augment the pharmacological results we observed.

To conclude, this research demonstrated through extensive *in vitro* and *in vivo* studies that USP36 is an oncogene in colon cancer through targeting c-Myc and uncovered CBF as a promising therapeutic agent for colon cancer.

## MATERIALS AND METHODS

### Bioinformatic analysis

Gene Expression Profiling Interactive Analysis (GEPIA; http://gepia.cancer-pku.cn/) [40], the online tool, was applied to perform the differential analysis of USP36 RNA expression between normal and colon cancer based on TCGA and GTEx data. In addition, the relation of USP36 expression with different stages of colon cancer, overall survival (OS) analysis, as well as disease-free survival (DFS) analysis, were also performed.

### Cell culture and oxaliplatin (Oxa)-resistant cell line establishment

Four colon cancer cell lines, which included SW480, HCT116, CaCO2, and Lovo, were obtained from ATCC (VA, USA), and were grown in RPMI 1640 medium (10% FBS and 1% penicillin-streptomycin). Cells were cultivated in a humidified environment of 5% CO_2_ at 37°C.

Colon cancer OVA-resistant cell lines were established based on SW480 and HCT116 cell lines as Sun et al. recently described [41]. Briefly, parental SW480 and HCT116 cells were treated with the intermittently and gradually increasing dose (0.5, 0.6, 0.7, 0.8, ……, and 2 μM) of OVA, and the duration was two months. In the beginning, the parental cells were grown and passaged in the media containing 0.5 μM of OVA. A week later, the surviving cells were subsequently exposed to a higher dose of OVA for another week (the concentration of OVA was increased every week by 0.1 μM up).

### Cell transfections and treatments

By using Lipofectamine^®^ 3000 reagent (Invitrogen, MA, USA), cell transfection was carried out for 48 h as previously reported [42]. The efficiency of all transfections was examined by detecting both mRNA and protein levels with RT-PCR and western blot, respectively.

HCT116 cells divided into diverse groups were subjected to different treatments: EV group (cells transfected with empty vector (EV)), USP36 OE group (cells transfected with lentivirus vectors for DUSP36 overexpression (USP36 OE)), sh-NC group (cells transfected with irrelevant nucleotides to act as a negative control), sh1-USP36 group (cells transfected with a short hairpin RNA (shRNA)#1 that specifically targeted USP36), sh2-USP36 group (cells transfected with shRNA#2 that specifically targeted USP36 (sh2-USP36)), sh3-USP36 group (cells transfected with shRNA#3 that specifically targeted USP36), sh-NC + 0 μM CBF group (cells transfected with irrelevant nucleotides prior to treatment with 0.05% DMSO, the vehicle of Cinobufotalin (CBF)), sh-NC + 5 μM CBF group (cells transfected with irrelevant nucleotides prior to treatment with 5 μM CBF), sh-USP36 + 0 μM CBF group (cells transfected with sh2-USP36 prior to treatment with 0.05% DMSO), and sh-USP36 + 5 μM CBF group (cells transfected with sh2-USP36 prior to treatment with 5 μM CBF).

### RT-PCR

Using TRIzol reagent, Total RNA was extracted from colon cancer cells to generate cDNA for RT-qPCR analysis. Then, the cDNA was amplificated with QuantiTect PCR Kits (Qiagen, CA, USA) by utilizing a 7500 Real-Time PCR System (Applied Biosystems, CA, USA). The primer sequences for USP36 and GADPH were described as follows: USP36, reverse: 5′-CATCGACGCCATGCAGAAAG-3′, forward: 5′-AGTAGGGGTCGTAGGTGTCC-3′; GAPDH, reverse: 5′-GAGAAGGCTGGGGCTCATTT-3′, forward: 5′-AGTGATGGCATGGACTGTGG-3′. The calculation of relative RNA expression was based on the 2^−ΔΔCt^ method [43].

### Western blot

Western blot was performed as previously described [44]. In brief, after isolating total protein from tissues and cells with RIPA buffer (10X) (Cell Signaling, MA, USA), protein quantification was performed with a BCA assay kit (Sigma-Aldrich, MO, USA). The total protein was separated by SDS-PAGE gel, and subsequently transferred onto PVDF membranes. After blocking with skim milk, membranes were incubated with primary antibodies specific for USP36, CD133, CD44, Nanog, Oct-4, c-Myc, and GADPH overnight. Next, membranes were washed thrice prior to 2 h incubation with HRP-conjugated secondary antibody. At last, an ECL detection reagent (Sigma-Aldrich, MO, USA) was used to visualize protein bands. The information on antibodies used in this study was listed in Supplementary Table 1.

### Colony formation

HCT116 cells (approximately 500 cells) that received different transfection and/or treatments were seeded into six-well plates (Corning, NY, USA). After cultivation in a 5% CO_2_ incubator at 37°C for two weeks, cell colonies were fixed with 4% paraformaldehyde before a 10 min-staining of 1% crystal violet. The dishes were gently washed, counted, and photographed under a BX51 microscope (Olympus, Tokyo, Japan).

### Edu staining

EdU staining was usually used to analyzed the cancer cell proliferation [45]. Briefly, the EdU Staining Proliferation Kit was applied to assess the proliferative capability of HCT116 cells from different groups in line with the protocol provided by manufacturers. By using a BX51 microscope, images were obtained to observe and calculate EdU-positive cells.

### CCK-8 assay

CCK-8 assay was conducted to calculate the IC50 value to OVA of HCT116 cells from different groups [46]. In brief, HCT116 cells grown in 96-well plates were treated with a series of concentrations of Oxa (1, 2.5, 5, 10, 20, and 50 μM) for 24 h incubation. Afterward, 10% of CCK-8 solution was added for additional 1.5 h incubation. Finally, the absorbance (450 nm) was measured under a Microplate reader (Bio-Rad, CA, USA) for the calculation of IC50 value.

### Sphere formation assay

Because tumor sphere formation is correlated with the proportion of CSCs [47], tumor sphere formation was performed to evaluate the effects of diverse treatments on the proportion of CSCs *in vitro*. A density of 1,200 HCT116 cells from different groups was plated on each well of 6-well ultra-low attachment plates and grown in medium supplemented with insulin, basic FGF, and EGF (the concentration was 5 μg/mL, 20 ng/mL, and 20 ng/mL, respectively). After finishing a 2-week incubation, finally, the spheroid number with a diameter of more than 50 μm of each well was counted under a light microscope.

### Wound healing assay

Cell migration was assessed using a scratch wound healing assay following a previous study [48]. After finishing the transfection, a density of 1.5 × 10^5^ HCT116 cells was seeded into six-well plates and cultivated. After forming a monolayer of cells, a similar size of scratches was created in the cell layer of each well. Then, scratched cells were removed, and the remaining cells were treated with different agents and subjected to another 24 h cultivation. A BX51 microscope was utilized to photograph the same position of scratches at 0 and 24 h after scratching.

### Transwell assay

The invasion of HCT116 cells was evaluated by using Transwell chambers (Corning, NY, USA) [49]. Briefly, cells with or without transfection resuspended 200 μL DMEM with diverse agents were added into the upper chambers. Simultaneously, 700 μL DMEM with serum was added to the lower chamber. After 24 h incubation, cells still in the upper chamber were wiped, while cells traversing the membranes to the lower chamber were subjected to fixation with 4% paraformaldehyde prior to staining with 0.1% crystal violet. Finally, under a BX51 microscope, the stained HCT116 cells were imaged and counted (five random visual fields).

### Co-immunoprecipitation (Co-IP)

HCT116 cells were lysed and incubated with the specific antibody, or bead-conjugated FLAG or HA overnight at 4°C. Then, a lysis buffer was utilized to rinse beads coupling immuno-complexes prior to eluting precipitated proteins with SDS-PAGE buffer. Finally, the separation of eluted proteins was performed with SDS-PAGE gels. The interacting proteins were detected by western blot.

### *In vitro* ubiquitination assay

HCT116 cells that finished the transfection of HA-c-Myc combined with or without Flag-USP36, or the transfection of USP36 OE combined with or without CBF treatment were subjected to the administration of MG132 with a concentration of 10 μM for 6 h. Then, cells were harvested, lysed, and immunoprecipitated by c-Myc antibody or bead-conjugated HA. Finally, the precipitated proteins were exploited to detect the level of ubiquitination for c-Myc using western blot.

### Cycloheximide (CHX) chase assay

The CHX chase assay enables to observe degradation kinetics of proteins and therefore is a good tool to study protein stability [50]. HCT116 cells were transfected with EV/USP36 OE or treated with vehicle/CBF. Afterward, cells were cultivated in the medium containing 20 μg/mL of CHX. At 0, 4, 8, and 12 h, cells were collected for detecting the protein levels with western blot.

### Molecular docking

The structure of USP36 (Primary accession: B1AQJ2) and the molecular structure of CBF (Compound CID: 259776) were downloaded from the AlphaFold Protein Structure Database and PubChem, respectively. AutoDock tools were exploited for docking simulation of CBF and predicting its binding affinity with the USP36 [51]. The results were visualized by the PyMOL tool (Version 1.3, Schrödinger, LLC, NY, USA).

### Ubiquitin (Ub)-AMC hydrolysis assay

To investigate the effect of CBF on USP36 activity, Ub-AMC hydrolysis assay was carried out as Li et al. reported [52]. In brief, USP36 plus 50 nM Ub-AMC mixture was added to each well for 30 min incubation, and fluorescence levels were subsequently examined at Ex355/Em460. In addition, CBF was tested for the ability to quench the fluorescence of 50 nM AMC in the absence of enzyme. CBF was then tested in dose-response from 0.5 μM to 8 μM to determine *in vitro* IC50 values.

### *In vivo* animal studies

All animal procedures in this study were approved by the Institutional Animal Care and Use Committee of The Second Xiangya Hospital (Approval no. 20220646). A total of 120 BALB/c nude male mice (18–22 g, 6–8 weeks) were purchased from Hunan Experimental Animal Center. All mice were maintained under SPF conditions and subjected to one-week acclimation prior to the following experiments.

Initially, HCT116 cells were stably transfected with either EV or USP36 OE plasmids before subcutaneous injection into mice. To investigate the *in vivo* tumor-initiating capacity of USP36, 1 × 10^3^, 1 × 10^4^, and 1 × 10^5^ of USP36-overexpressing HCT116 cells were respectively injected into the flanks of mice (*n* = 6/group). The EV-transfected HCT116 cells served as the control. After growing for six weeks, tumors were collected to calculate the tumor-initiating cell (TIC) frequency with the Extreme Limiting Dilution Analysis (ELDA) software [53].

Next, to determine the effect of USP36 or CBF on the growth of colon tumors, mice were randomly divided into eight groups (*n* = 6 per group): EV+vehicle, EV+OXA, USP36 OE+vehicle, USP36 OE+Oxa, control, Oxa, CBF, and Oxa +CBF groups. Mice from both the EV+vehicle and EV+OXA groups were injected subcutaneously into their right flanks with 5 × 10^5^ HCT116 cells with EV transfection, and exposed to Oxa (7.5 mg/kg) [54] or saline biweekly after the tumor reach 30–40 mm^3^. Meanwhile, mice from the USP36 OE+vehicle and USP36 OE+ Oxa groups were injected with 5 × 10^5^ USP36-overexpressing HCT116 cells and then exposed to the same administration. The same density of HCT116 cells was injected subcutaneously into the right flanks of mice from the control, Oxa, CBF, and Oxa+CBF groups. After the tumor reached 30–40 mm^3^, mice were exposed to vehicle, 7.5 mg/kg Oxa, 250 mg/kg CBF (dissolved in saline with 0.05% DMSO) [55], and the combination of Oxa and CBF. On day 7, the length and width of tumors were monitored with ruler once a week for four weeks and the tumor volume was calculated using the following equation: Tumor volume = (length × width^2^)/2, and the growth curve of tumors was plotted. At the end of the experiment, the surviving mice were sacrificed in a humanitarian way.

For patients with colon cancer, the liver is the most common metastatic site. To study metastasis of colon cancer cells to the liver, the remaining mice were divided into six groups defined as follows: EV, USP36 OE, control, Oxa, CBF, and Oxa+CBF groups. HCT116 cells with EV or USP36 OE transfection were injected into the portal vein of the liver of mice from the EV and USP36 OE groups, respectively. HCT116 cells without transfection were injected into the portal vein of the liver of mice from control, Oxa, CBF, and OVA+CBF groups. After finishing the treatment (day 28), liver tissues were dissected and harvested to investigate liver metastasis with H&E staining.

### Statistical analysis

Data were expressed as the mean ± SEM from at least three different experiments and analyzed with GraphPad Prism 8.0.1 version software (GraphPad Software, CA, USA). To determine whether there are significant different among groups, a one-way analysis of variance followed by Tukey’s post hoc test was performed; and *P* < 0.05 represented that the difference was statistically significant.

## Supplementary Materials

Supplementary Figures

Supplementary Table 1
