# Supplementary material for: Cinobufotalin regulates the USP36/c-Myc axis to suppress malignant phenotypes of colon cancer cells in vitro and in vivo
Source: Aging (Albany NY). 2024 Mar 15;16(6):5526–44. doi: 10.18632/aging.205661 (PMC11006458; doi:10.18632/aging.205661)
Supplement: Supplementary Figures [file aging-16-205661-s001.pdf]

SUPPLEMENTARY FIGURES

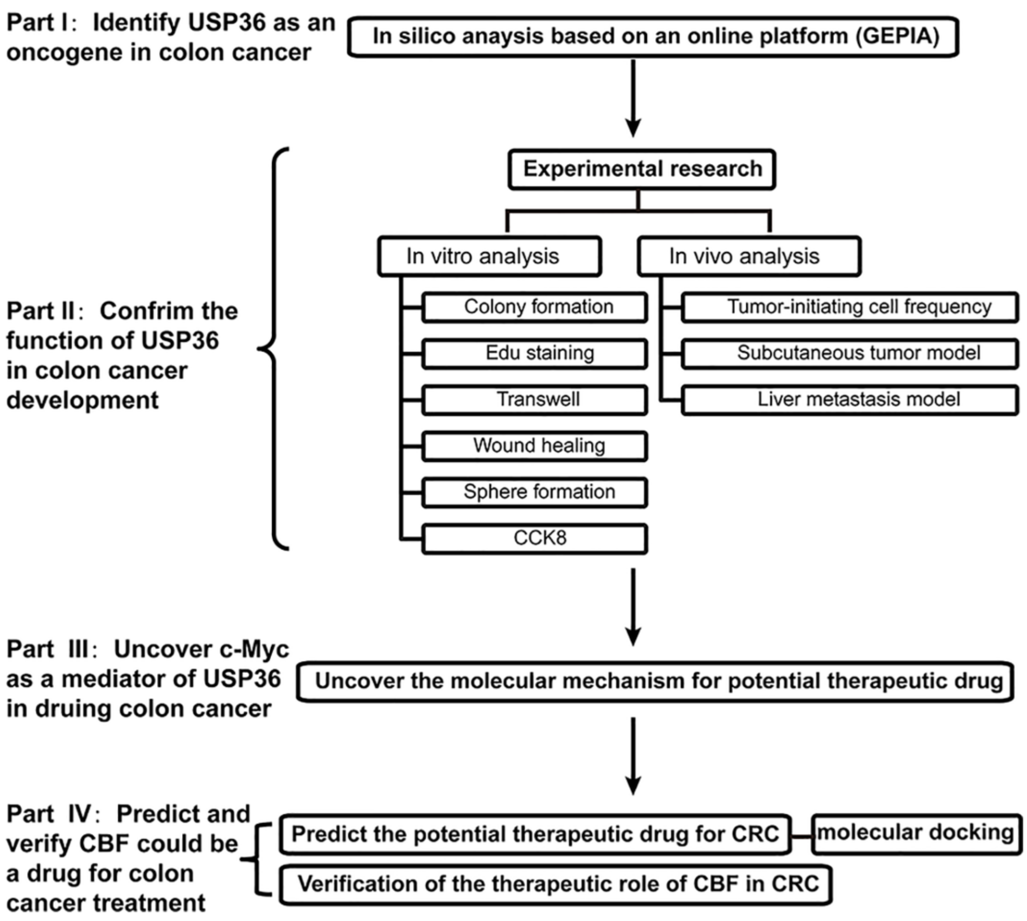

Supplementary Figure 1. A flow chart of experiments in this study.

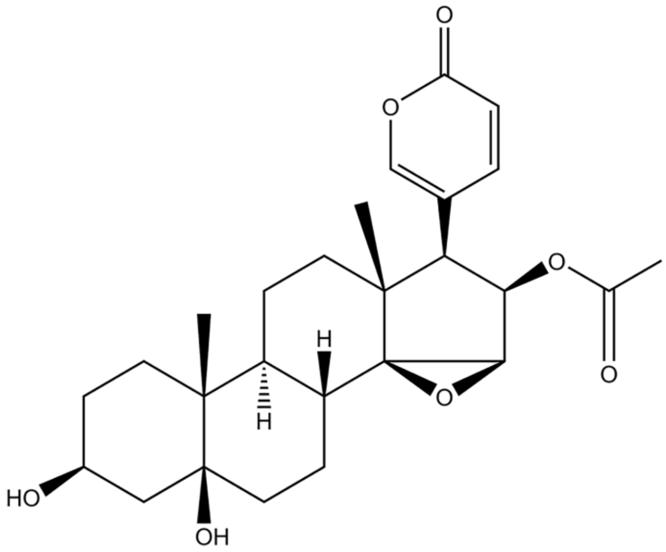

Supplementary Figure 2. Chemical structure of CBF.
