# Supplementary material for: Cinobufotalin regulates the USP36/c-Myc axis to suppress malignant phenotypes of colon cancer cells in vitro and in vivo
Source: Aging (Albany NY). 2024 Mar 15;16(6):5526–44. doi: 10.18632/aging.205661 (PMC11006458; doi:10.18632/aging.205661)
Supplement: Supplementary Table 1 [file aging-16-205661-s002.pdf]

SUPPLEMENTARY TABLE

Supplementary Table 1. The antibodies used in this research.

Antibody	Manufacturer	Cat.no
USP36	Proteintech Group Inc.	14783-1-AP
CD133	Bioss Inc.	bs-4770R
CD44	GeneTex	GTX102111
Nanog	GeneTex	GTX627421
Oct-4	GeneTex	GTX101497
c-Myc	GeneTex	GTX103436
GAPDH	GeneTex	GTX100118
